# Biomechanics of wheelchair turning manoeuvres: novel insights into wheelchair propulsion

**DOI:** 10.3389/fspor.2023.1127514

**Published:** 2023-06-13

**Authors:** Dhissanuvach Chaikhot, Matthew J. D. Taylor, W. H. K. de Vries, Florentina J. Hettinga

**Affiliations:** ^1^ Department of Physical Therapy, Christian University of Thailand, Nakhon Pathom, Thailand; ^2^ School of Sport, Rehabilitation and Exercise Science, University of Essex, Colchester, United Kingdom; ^3^ Swiss Paraplegic Research, Shoulder Health & Mobility Group, Nottwil, Switzerland; ^4^ Department of Sport, Exercise and Rehabilitation, Northumbria University, Newcastle Upon Tyne, United Kingdom

**Keywords:** wheeled mobility, locomotion, upper body exercise, physical activity, propulsion biomechanics, handrim, manoevrability, dayly activity

## Abstract

**Introduction:**

Wheelchair turning biomechanics is an under researched area despite its obvious relevance to functional mobility of wheelchair users. Wheelchair turns might be linked to a higher risk of upper limb injuries due to the increased forces and torques potentially associated with asymmetric movement. Our aim was to obtain a better theoretical understanding of wheelchair turning by biomechanically analyzing turns compared to steady-state straightforward propulsion (SSSFP).

**Methods:**

Ten able-bodied men received 12-min familiarization and 10 trials (in a random order) of SSSFP and multiple left and right turns around a rectangular course. A Smart^wheel^ was mounted at the right wheel of a standard wheelchair to measure kinetic parameters during SSSFP and of the inner hand during right turns and the outer hand during left turns. A repeated measures ANOVA was used to detect differences across tasks.

**Results:**

Two strategies were identified: 3% demonstrated roll turns and 97% spin turns. Spin turns consisted of three phases: approach, turning and depart phase. The turning phase was accomplished by increasing peak force (72.9 ± 25.1 N vs. 43.38 ± 15.9 N in SSSFP) of the inner hand, while maintaining high push frequency of the outer hand (1.09 ± 0.20 push/s vs. 0.95 ± 0.13 push/s in SSSFP). Peak negative force and force impulse during the turning phase were much higher than SSSFP, 15.3 ± 15.7 and 4.5 ± 1.7 times higher, respectively.

**Conclusion:**

The spin turn strategy might carry an increased risk of upper limb injuries due to higher braking force and requires particular attention by rehabilitation professionals to preserve upper limb function of long-term wheelchair users.

## 1. Introduction

Manual wheelchair users need to negotiate their environment and will face daily physical barriers such as curbs, slopes, obstacles, uneven terrains (1) requiring a variety of daily propulsion demands such as changing direction while moving forward (2, 3). Sixty-three percent of real-life propulsion bouts are dominated by slow and short-changes in wheelchair speed and direction (4). These typical bouts may require a higher pushing force compared with steady-state straight forward propulsion (SSSFP) on a smooth surface floor (5, 6). The higher peak forces and torques during propulsion could lead to a high risk of upper limb injuries in manual wheelchair users (7), with the prevalence of upper limb pain ranging from 55%–72% (8–10).

Wheelchair users perform approximately 900 turns (moving turns and turn-on-the-spot) per day which equals a turn every 3.6 m (11). Thus changing/adjusting direction is fundamental to negotiating the environment and wheelchair use. Despite the common encounters with barriers, the prevalence of turning and the highly potentially injurious nature of the activity, the biomechanics of these maneuvers is still a relatively new area of research. For example, Rouvier et al. (1) reviewed the literature assessing the biomechanics of wheelchair users encountering barriers. Ascending a slope was the most studied scenario, while cross-slopes and curbs (ascent) were scarcely studied despite the specific propulsion strategies needed for these. Furthermore, Rouvier et al. (1) suggested a task analysis should be undertaken by separating start-up, propulsion, braking, and turning.

The biomechanics of wheelchair turning is also, relatively, scarcely studied. The current literature has focused on figure-of-eight turning (5, 12, 13), turning 360° on the spot, turning around a 2 m radius circle, or slalom course turning (14), and using mechanical jigs/robotics to test wheelchair configurations while turning (15–17). However, there does not appear to be any published studies which have investigated sharp turning when propelling a wheelchair, and what strategies are used to turn through 90° – thus replicating a maneuver used when entering rooms. Therefore, a study providing theoretical insights into wheelchair turning is needed.

In human walking gait, turning maneuvers have been explored, and two main turning strategies were identified: spin and step turns (18). A turn towards the same side as the stance limb has been commonly referred to as a spin turn. Whereas the step turn is a turn away from the stance limb, e.g., land on the right leg and turn to the left. The step turn, a simpler turning strategy, may offer advantages over the spin turn (18). Similarly, exploring turning strategy in wheelchair propulsion can be even more interesting as this functional mobility is new to almost everyone, and using the upper limbs for propulsive purposes provides an additional challenge to the upper limbs. Wheelchair turns might be linked to a higher risk of upper limb injuries due to the increased forces and torques potentially associated with this asymmetric movement. Theoretical insights in wheelchair turns could improve the wheelchair turning instruction during early rehabilitation to preserve upper limb function of long-term wheelchair users. However, wheelchair turning strategies have not yet been studied nor identified. The present study aimed to obtain better theoretical understanding of wheelchair turning by describing 90° turning maneuvers and assessing inner and outer push characteristics in approach, execution and depart phase. The secondary aim was to compare the timing parameters and force requirements of turns to those demonstrated in SSSFP. It was hypothesized that due to the asymmetric nature of changing direction, forces would be higher than seen in SSSFP. To better understand the basic turning movement without potential disabilities impacting on the turning biomechanics, we focused on able-bodied propulsion first.

## 2. Methods

### 2.1. Participants

Ten able-bodied young men participated in this study (26 ± 5 years, 1.73 ± 0.07 m, body mass: 69 ± 10 kg). The participants were recruited using volunteer and convenience sampling methods. All participants gave written informed consent prior to participation. Ethical approval for this study was obtained from the University of Essex Ethics Committee.

### 2.2. Design and experimental protocol

To investigate the forces and torques during turning maneuvers, participants performed standardized propulsion activities under experimental conditions in an instrumented wheelchair (Smart^wheel^ 3 Rivers Holdings, Mesa, AZ). Both 3-dimensional forces and moments (Smart^wheel^) and 3-dimensional kinematics (Vicon) of the upper extremity were evaluated in each maneuver. Prior to the data collection session, participants familiarized themselves with the experimental protocol by propelling the wheelchair at comfortable speed around a rectangular course (6 m × 4 m) delimited by cones. The familiarization sessions consisted of four 3-minute practice blocks with 2-minute rest in between as described by a previous study (19).

The testing maneuvers consisted of 3 tasks based on the Wheelchair Skills Test (WST version 4.2 manual), which are often encountered during daily life:

1) Steady-state straight forward propulsion (SSSFP)
2) turning 90° to the right while moving, measuring the inner hand (TR_i_)
3) turning 90° to the left while moving, measuring the outer hand (TL_o_)

Ten trials of each task were taken with a 1-minute break between trials (20). The order of testing maneuvers was randomized. For SSSFP, a 12-meter prescribed pathway was labeled on the smooth laboratory floor to guide participants to roll in a straight line in 10 s from one end to the other end of the room. For TR_i_ and TL_o_, participants were asked to roll a wheelchair at comfortable speed along the pathways marked by yellow cones (19 cm wide, 5 cm high) and colored tape on the floor. The square turning tasks consisted of rolling the wheelchair 3-meters in a straight line (SSSFP), then turning 90° (∼1-meter radius marked by a 23-cm high orange cone) to the right or left. We have intentionally focused on specifying the turn angle at 90° to allow the participants to choose their turning strategies, while not constraining participants to vary the turning radius. This is to mimic real-life situations where wheelchair users will have to turn in for example corridors, with no further guidance specified. Kinetic data were always measured at the right wheel, which is the inner wheel during a right turn or the outer wheel during a left turn.

### 2.3. Biomechanics measurement

All manual wheelchair maneuvers were performed in a standardized wheelchair. A non-folding ultra-light wheelchair (Quickie, USA) (seat height above the ground: 0.50 m; the diameter of the wheels: 0.64 m; chair width: 0.42 m; chair depth 0.40 m; 14-kg total mass) was mounted with a force- and torque-sensing Smart^wheel^ (3 Rivers Holdings, Mesa, AZ) to the right wheel to collect kinetic data (wheel diameter of 0.64 m and hand-rim diameter of 0.56 m), opposite the left wheel of identical size to maintain symmetry. Kinetic data were collected at 240 Hz and digitally filtered with 8th Butterworth low-pass filter and 20 Hz cutoff frequency. The characteristics and properties of the Smart^wheel^ are described in more detail elsewhere (21). Peak tangential force (Ft) and peak negative tangential force (Ft_neg_), peak torque (Mz or torque around the wheel hub which is responsible for angular acceleration of the wheel) and peak negative torque (Mz_neg_) and push characteristics (speed, push angle, push frequency, push time and cycle time) of each trial were collected. Ft was calculated from Mz, (Mz divided by rim radius) and therefore contains the same amount of information. Three stable consecutive push cycles of SSSFP and an approach push, turning push, depart push of right and left turns were used for processing and analysis. Ft was chosen to indicate the effort required during turning compared to SSSFP. Ft_neg_ reported in this study refers to the braking/deceleration force (22). Force impulse was calculated over the duration of the selected push phase.

Right and left turns were measured at the right wheel, which was the inner wheel during turning right and the outer wheel during turning left. The turning pivot was marked by a high orange cone on the rectangular route. Hand movements were identified by using 3-dimensional kinematics with a 7 Camera Vicon system (Oxford Metrics Ltd., UK). Reflective markers were placed on the third metacarpophalangeal joint (3rd MCP), radial styloid, ulnar styloid, lateral epicondyle on both sides of upper extremities, and hub of the right wheel. The 3-D data were collected at 100 Hz and digitally filtered with 4th order butterworth low-pass filter, 0-lag and 20 Hz cutoff frequency (23).

### 2.4. Statistical analysis

Data were analyzed using SPSS. Descriptive statistics were used to describe the demographic profiles of the participants. Data of ten trials per participant (of each task) were averaged. Comparative statistical analyses for force impulse and timing parameters across 7 conditions of the 3 tasks (SSSFP and 3 phases of TR and TL) were employed by a repeated measure analysis (ANOVA) with Bonferroni post-hoc test and adjusted for multiple comparisons to identify which condition was significantly different from each other. The level of significance was set at *p* < 0.05.

## 3. Results

### 3.1. Description of a turn

**Figure 1:**
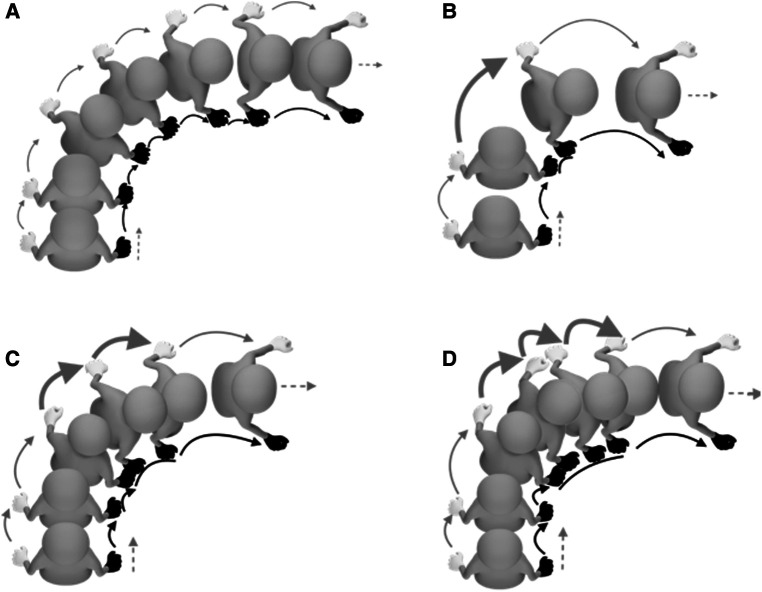
Approximate hand movements during 90° turns to the right. (**A**) RT was achieved by propelling the wheelchair with both hands in the new direction synchronously. A black hand indicates right hand (inner hand), white hand indicates left hand (outer hand). Dashed gray arrows indicate direction of travel, thin black and gray arrows indicate approach pushes and depart pushes of the right and left hand, respectively. Thick black and gray arrows indicate the turning push of the right and left hand, respectively. Spin turn was executed by braking with the inner hand (black solid lines indicate braking periods) whilst the outer hand changed direction by spinning the wheelchair around the inner wheel by increasing the push frequency. Spin turn was sub-categorized by numbers of spinning pushes of the outer hand: (**B**) 1, (**C**) 2 or (**D**) 3 pushes.

Observed hand movements determined the turning types. There were 2 types of 90° turns identified: a roll turn (RT) and a spin turn (ST) (Figure 1). RT was achieved by propelling the wheelchair with both hands in the new direction synchronously, see Figure 1A. It was found in only 2 and 4 trials during turn right and left respectively.

ST was executed by braking with the inner hand whilst the outer hand changed direction by spinning the wheelchair around the inner wheel, see Figures 1B–D. Both types of turns consisted of three phases: approach, turning, and depart phase. ST was performed predominantly in both right and left turns, 98 and 96 out of 100 trials respectively. ST was sub-categorized by the number of spinning pushes of the outer hand: either 1, 2 or 3 spinning pushes. Two-spinning push turns were used predominantly in turn right and left, 74% and 90% of total trials respectively.

**Figure 2:**
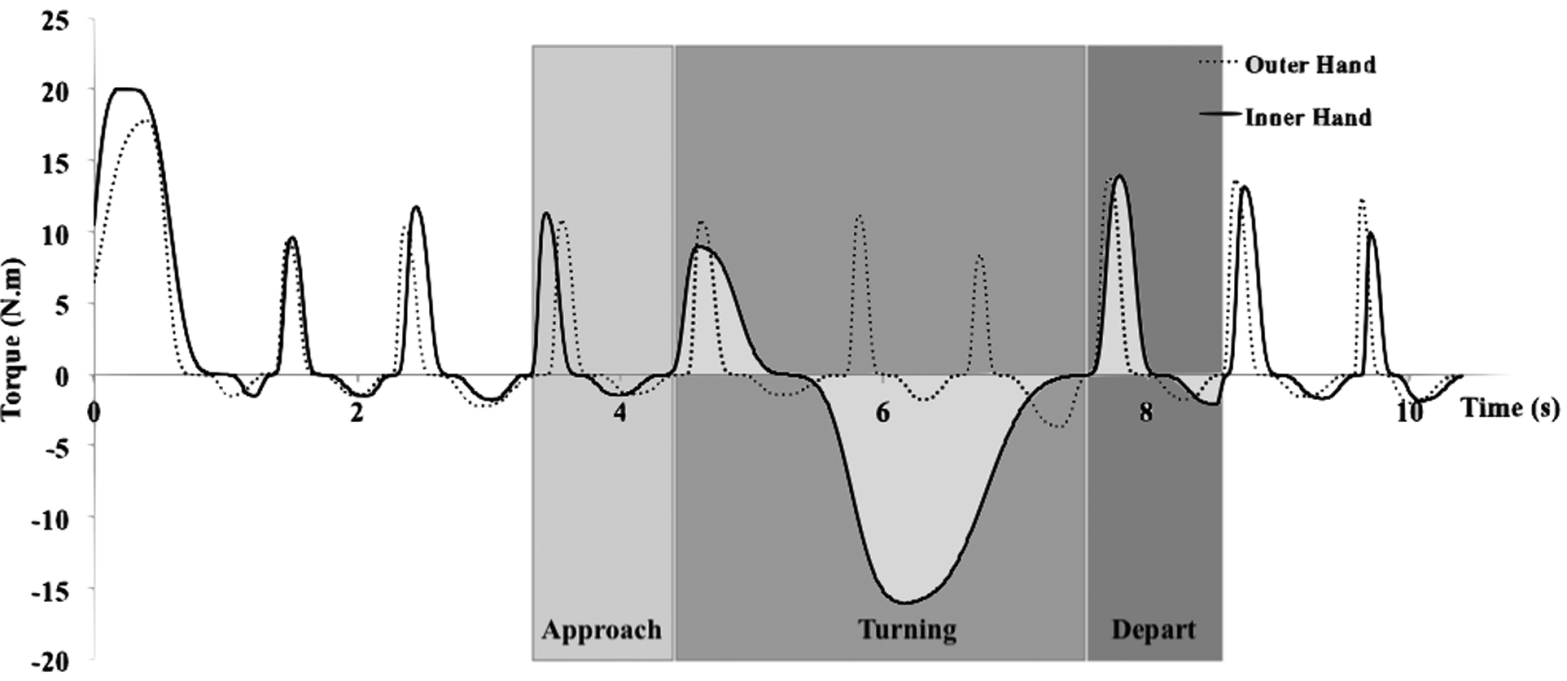
Typical pattern of torque during a 2-spinning push turn to the right across time. A solid line indicates torque of the inner wheel (TR_i_). A dashed line indicates torque of the outer wheel (TL_o_).

In a ST to the right, the turning phase was defined by the push cycle in which the Mz reached the lowest negative value (braking force) (solid line in Figure 2). When turning left, the braking event (at the left wheel) started at the time where the trajectory of the left elbow marker approached the cone marker on the floor and reached the uppermost in z-axis (superior-inferior position) and ended where the trajectory started to decline to the lowest point. The push cycles (at the right wheel) that simultaneously occurred during this braking event were defined as the turning phase of the left turn (dashed line in Figure 2). The adjacent push before and after were defined as the end of the approach and the start of the depart phase, respectively, in both right and left turns.

### 3.2. Speed

The average speed of SSSFP (0.98 ± 0.17 m/s) was faster than the average speed of turning right (0.55 ± 0.08 m/s) (*p* < 0.001) and turning left (0.69 ± 0.08 m/s) (*p* < 0.001). Due to measurement setup with only one Smart^wheel^ on the right side, the measured speed of a left turn was faster (Smart^wheel^ being the outer wheel in turning) than a right turn (Smart^wheel^ being the inner wheel during turning) (*p* < 0.001). The comparison of speed for SSSFP and the different phases of turning left and turning right is shown in Table 1.

**Table 1:** Mean values (±SD) and statistical comparisons of force impulse and timing parameters across the 3 tasks.

Task	Impulse [N.s]*	Speed [m/s]*	Push angle [°]*	Push frequency [pushes/min]*	Push time [s]*	Cycle time [s]*
SSSFP	9.27 ± 3.25^§^	0.98 ± 0.17	49.17 ± 8.88	0.95 ± 0.13^§^	0.28 ± 0.03^§^	1.10 ± 0.16^§^
TR_i_ Approach	11.11 ± 6.27^§^	0.78 ± 0.1^†^	45.05 ± 11.01	0.97 ± 0.14^§^	0.35 ± 0.12^§^	1.07 ± 0.15^§^
TR_i_ Turn	38.39 ± 11.07	0.45 ± 0.08^†‡^	36.35 ± 7.38^†^	0.34 ± 0.08	0.82 ± 0.25	3.14 ± 0.67
TR_i_ Depart	20.11 ± 9.59^§^	0.44 ± 0.10^†‡^	31.00 ± 10.94^†^	1.00 ± 0.21^§^	0.54 ± 0.22	1.09 ± 0.19^§^
TL_o_ Approach	11.32 ± 4.95^§^	0.73 ± 0.11^†§¶^	41.55 ± 7.89^¶^	0.96 ± 0.19^§^	0.38 ± 0.13^§^	1.23 ± 0.29^§^
TL_o_ Turn	12.01 ± 4.92^§^	0.65 ± 0.07^†‡§¶^	41.80 ± 6.90^¶^	1.09 ± 0.16^§^	0.35 ± 0.09^§^	1.00 ± 0.16^§^
TL_o_ Depart	14.59 ± 4.63^§^	0.69 ± 0.07^†‡§¶^	46.48 ± 9.05^§¶^	1.04 ± 0.18^§^	0.39 ± 0.12^§^	1.01 ± 0.14^§^

* Significant main effect for Task.

^†^ The value is different from SSSFP.

^‡^ The value is different from TR_i_ Approach.

^§^ The value is different from TR_i_ Turn.

^¶^ The value is different from TR_i_ Depart. SSSFP, steady-state straightforward propulsion; TR_i_, inner hand of turn right; TL_o_, outer hand of turn left. All differences are significant at *p* < 0.05.

### 3.3. Peak force and brake

**Figure 3:**
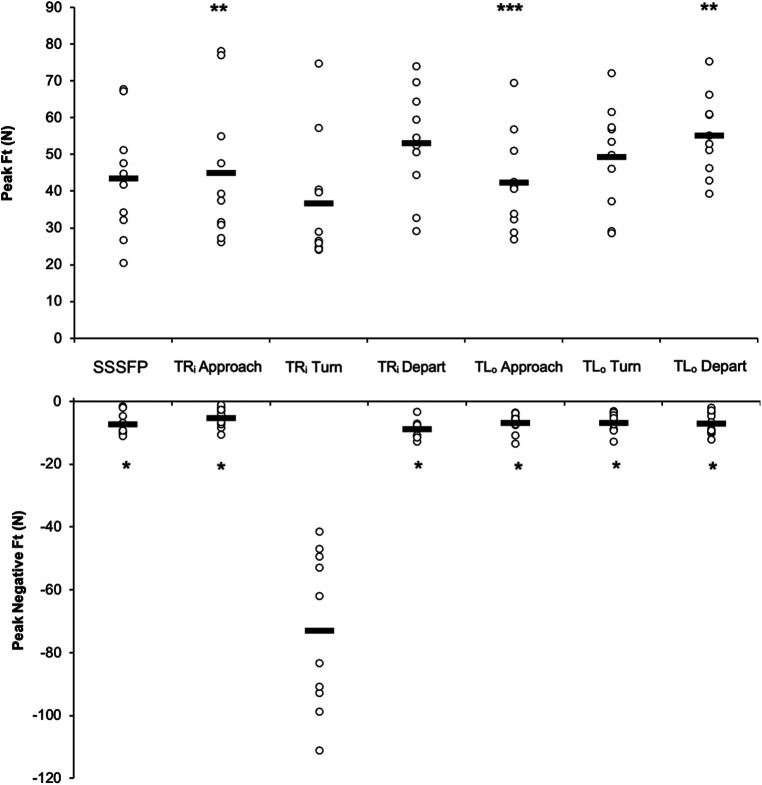
Comparisons of peak tangential force (Ft) and peak negative tangential force (Ft_neg_) across tasks. SSSFP, TR_i_ inner hand of turn right, TL_o_ outer hand of turn left. *Value different from the Ft_neg_ turning phase of TR_i_. **Value different from the Ft turning phase of TR_i_. ***Value different from the Ft depart phase of TL_o._

Comparisons of Ft and Ft_neg_ during the three propulsion tasks are presented in Figure 3. There was a significant task effect (*p* < 0.001) for Ft across the propulsion tasks. Ft during the turning phase of turning right was significantly lower than during the approach phase of turning right (*p* = 0.044) and depart phase of turning left (*p* = 0.009). In turning left, Ft of the approach phase was significantly lower than during depart phase (*p* = 0.031). Ft_neg_ during the turning phase of turning right was significantly higher than during all phases of turning right, turning left and SSSFP (*p* < 0.001). There was a significant task effect (*p* < 0.001) for Ft_neg_ across the propulsion tasks. During the turning phase, the inner hand applied a peak negative force 15.3 ± 15.7 times higher than the peak negative force of SSSFP. The (negative) force impulse during the turning phase was higher than all the phases of turning tasks and SSSFP (*p* < 0.001), as shown in Table 1. This force impulse was 4.5 ± 1.7 times higher than SSSFP.

### 3.4. Timing parameters

Speed of SSSFP was faster than the approach, turning and depart phase of turning right and left (all *p* < 0.001). When turning right the approach phase was faster than turning and depart phases (*p* < 0.001). When turning left, there were no differences in speed across the 3 phases. The push angle of SSSFP was greater than the turning and depart phase of turning right (*p* = 0.002 and *p* = 0.008, respectively). The push angle during the depart phase of turning left was greater than the depart phase (*p* = 0.015) and turning phase (*p* = 0.022) of turning right. Push frequency during turning phase of turning right was lower than all the phases of turning right (*p* < 0.001), turning left (*p* < 0.001) and SSSFP (*p* < 0.001). Push time and cycle time during turning phase of turning right were different from SSSFP (*p* = 0.002), the approach, turning and depart phase of turning left (*p* = 0.002, 0.009 and 0.009, respectively). The mean values with standard deviations of timing parameters and statistical comparisons between SSSFP and the turning tasks are presented in Table 1.

## 4. Discussion

The present study offers a first theoretical insight during wheelchair turning maneuvers and provides detailed biomechanical information on the push characteristics and peak forces and torques required to complete 90° turns. Two turning strategies were identified, ST and RT. ST was an asynchronous turning pattern accomplished by a deceleration, initiated in approach phase, accompanied by an increasing braking force of the inner hand and a higher push frequency of the outer hand during turning phase. This strategy was used 97% during turns. Based on the biomechanical analysis three phases were identified: approach, turning, and depart. These data help us to understand asymmetrical wheelchair turning movements and to better interpret how these maneuvers could lead to higher risks for upper body injuries due to the associated high peak loads.

The results clearly showed that ST was the most preferred turning strategy in novice users. This might imply that ST is the most effective turning strategy, which is simplest to perform in 90° turns. In most trials, asymmetric hand patterns were seen, in which a turn was completed with a fixed-inner hand and 2 spinning pushes from the outer hand. This specific hand pattern may be related to the participant's characteristics and preference as well as the simplest turning strategy to be performed by novice users. Since RT was only used for 6 trials, we cannot compare to ST.

In daily living, turns are common for wheelchair users, and the median turn angle reported (39°, IQR: 24–67°) (11) is less than that used in this current work (90°). The turn angle we used was to replicate a turn into a room or corridor. Future work may want to investigate the biomechanics of turning at different angle/radii, including turning-on-the-spot (which is also a common turning maneuver) (11), to gain a more comprehensive overview of direction change and turning strategies in wheelchair propulsion. Furthermore, a more detailed biomechanical analysis is required to better understand what the best turning strategy is to reduce shoulder load, and thereby eventually reduce the likelihood of injury.

SSSFP speed was comparable to that reported in other studies using non-wheelchair users (24). This speed was faster than that seen during daily activities of wheelchair users where a median bout lasts for 21 s and travel 8.6 m at 0.43 m/s (4). This difference can be explained by the longer bout time than that used in this present study. The speed decreases upon approach in preparation for the turn. Since the approach was 3 m in length it is possible that this reflects a reduced push-off/acceleration from standstill in anticipation of the turn. Turn speed was slower than approach speed for both inside and outside turns. In addition, departure was also slower than approach, a consequence of the reduction in speed during the turn suggests that the participants were still accelerating at this stage as they had not reached approach speed or SSSFP speed. Future work may wish to look at more cycles of the approach and departure to establish the deceleration and acceleration strategies.

Togni et al. (11) reported turning during daily living turns were performed with a mean velocity (for all turns) of 0.57 ± 0.14 m/s and a mean turn radius, of 1.39 ± 0.20 m. In addition, a mean velocity of 0.36 m/s was reported for a turn radius of 0.6–0.8 m. These velocities are comparable to the current work and the 1-m turn radius used. The pattern of change in velocity from approach-turn-departure was comparable to that reported by Hwang et al. However, there are also some nuances. For example, Hwang et al. instructed their participants to propel, along a figure-of-eight path, at a self-selected maximum velocity, and as such approach speed (2.11 ± 0.43 m/s) was greater than that compared to this current work. The speed difference between left and right turns is because the measurement wheel was always mounted on the right side which is the inner wheel for right turn (less angular velocity) than the left/outside wheel. A similar pattern was also noted by Hwang et al. (5).

A faster approach speed indicates a better maneuverability and mobility during straightforward propulsion (25). However, this might not necessarily be the case for an asynchronous turn, which involves braking maneuvers. In straight-line trajectories, an increased speed of the driving wheels reduces resistive force losses, whereas there is an increase in resistive force losses during turning (5). A decrease in resistive force losses would decrease energy expenditure for a given task (26–28). In addition, when turning with increased speed, rotational inertia significantly increases torque required to accelerate a wheelchair (15). As a result, turning speed was lowered to reduce inertia and resistive force losses, resulting in reduction in force and torque required.

During the turning phase, the wheelchair was driven slowly by the outer hand while the inner hand generated a braking force to make a turn. This braking force was 15.3 ± 15.7, 20.2 ± 14.0 and 9.6 ± 6.0 times higher compared to braking force of SSSFP, approach, and depart phases, respectively. Even though speed preceding the turn in this study was markedly lower (comfortable speed: 0.78 ± 0.10 m/s) than that reported by Hwang et al. (maximum speed: 2.11 ± 0.43 m/s) (5), the magnitude of the braking force during turning (−0.90 ± 0.34 N/kg, normalized for all participants and averaged) was comparable to Hwang et al. (−1.03 ± 0.25 N/kg) (5). In real-life propulsion, a lot of turns are performed throughout a day with median of 913 ± 214 turns per day (11), thus emphasizing the potential risk of muscle fatigue and upper limb injuries caused by turning maneuvers in daily activities. In a day, manual wheelchair users travel in short bouts and frequent changes in speed and direction (3). The higher the peak force used within a bout of propulsion, the higher the cumulative energy will be that is required throughout the day. Boninger et al. (7) suggested that diminishing the occurrence of upper limb injuries could be achieved by reducing the force to around 5% of body weight during self-propulsion. Therefore, wheelchair spin turners might need to reduce the braking forces by minimizing speed changes during a turn (7).

There are limitations that need to be addressed. Firstly, all kinetic data were collected unilaterally where propulsion asymmetries may have gone unnoticed. The use of the same standardized ultra-light wheelchair to eliminate any bias caused by wheelchair model/setups, however, can limit the applicability of the results and indicates the need to study more wheelchair turning in more wheelchair designs and configurations. Conform Rouvier et al., it is recommended in future studies to carefully report wheelchair configuration and environmental characteristics (1), but standardized reporting methodologies have to be developed (29). Also, it will be important to report the speed of the wheelchair reference frame (generally centered between rear wheel center) in addition to the speed at the wheel as reported in the present study. Indeed, in straightforward propulsion, these speeds would be identical, however, this is not the case during turning. Finally, able-bodied participants were chosen to understand turning in a homogenous group of participants unaffected by different disabilities. Though it improves our understanding of the turning movement, it might limit the transferability to wheelchair users, and more research is needed on wheelchair propulsion in individuals with different disabilities. However, able-bodied individuals are to some extent comparable with newly injured individuals with intact upper body function (23). These findings are thus particularly applicable to the novice wheelchair population with intact upper body function. Lastly, it has to be mentioned that we have not included female participants in the current study. The low number of female participants has been raised as an issue in sport and exercise science (30), and differences in propulsion characteristics between sexes have been observed (31). It is therefore important to include more female propulsion data in future studies.

In conclusion, this study was the first to explore freely chosen turning strategies and address turning biomechanics in 3 phases during a 90° turn when propelling at comfortable speed, which helps us to understand asymmetrical wheelchair turning movements. Two turning strategies (ST and RT) were identified in this study, in which ST was dominant. This asynchronous turning strategy might carry an increased risk of upper limb injuries due to 15-times higher braking force of the inner hand during turning, compared to straightforward propulsion. This needs to be taken into consideration to preserve the upper limb function of long-term wheelchair users, especially since turning is executed repeatedly throughout the day. To minimize braking forces during turns, skill training by rehabilitation professionals focusing on decelerating the approach speed and reducing speed loss between the approach and turning phase is advised in an early stage.

## Data Availability

The raw data supporting the conclusions of this article will be made available by the authors, without undue reservation.
